# Association between the anion-gap and 28-day mortality in critically ill adult patients with sepsis: A retrospective cohort study

**DOI:** 10.1097/MD.0000000000039029

**Published:** 2024-07-26

**Authors:** Zeying Lou, Fanghua Zeng, Wenbao Huang, Li Xiao, Kang Zou, Huasheng Zhou

**Affiliations:** a Internal Medicine, The Second Hospital of Xingguo County, Ganzhou City, Jiangxi Province, China; b Department of Critical Care Medicine, The Second Hospital of Xingguo County, Ganzhou City, Jiangxi Province, China; c Department of Rehabilitation Medicine, The First Affiliated Hospital of Gannan Medical University, Ganzhou City, Jiangxi Province, China; d Department of Critical Care Medicine, The First Affiliated Hospital of Gannan Medical University, Ganzhou City, Jiangxi Province, China.

**Keywords:** anion gap, mortality, sepsis

## Abstract

Metabolic acidosis is usually associated with the severity of the condition of patients with sepsis or septic shock. Serum anion gap (AG) is one of the indicators of response metabolism. This study was performed to investigate whether the initial serum AG is associated with the 28-day mortality in critically ill adult patients with sepsis. This retrospective cohort study, a total of 15,047 patients with confirmed Sepsis disease from 2008 to 2019 from the Medical Information Mart for Intensive Care IV (MIMIC-IV) v1.0 database. The MIMIC-IV database is a comprehensive, de-identified clinical dataset originating from the Beth Israel Deaconess Medical Center in Boston, it includes extensive data on intensive care unit (ICU) patients, such as vital signs, lab results, and medication orders, spanning multiple years, accessible to researchers through an application process. AG can be obtained by direct extraction in the MIMIC-IV database (itemid = 50,868 from the laboratory events table of mimic_hosp), inclusion of AG values for the first test on first day of ICU admission. The patients were grouped into quartiles according to the AG interquartile range. The primary outcome was the 28-day mortality. Multiple logistic regression analysis was used to calculate the odds ratio (OR), while accounting for potential confounders, and the robustness of the results were evaluated in subgroup analyses. Among the 15,047 patients included in this study, the average age was 65.9 ± 16.0 years, 42.5% were female, 66.1% were Caucasian, and the 28-day mortality rate was 17.9% (2686/15,047). Multiple logistic regression analysis revealed the 28-day mortality in every increase of AG (per SD mEq/L), there is an associated 1.2 times (OR 1.2, 95% CI 1.12–1.29, *P* < .001) increase. Increased 28-day mortality (OR 1.53, 95% confidence interval 1.29–1.81, *P* < .001) in the group with the AG (15–18 mEq/L), and (OR 1.69, 95% confidence interval 1.4–2.04, *P* < .001) in the group with the highest AG (≥18 mEq/L), AG (<12 mEq/L) as a reference group, in the fully adjusted model. In adult patients with sepsis, the early AG at the time of ICU admission is an independent risk factor for prognosis.

## 1. Introduction

In the Third International Consensus Definitions for Sepsis and Septic Shock, sepsis is defined as a life-threatening organ dysfunction that is caused by a dysregulated host response to infection.^[1]^ Sepsis remains a common cause of death in critically ill patients. Patients with sepsis undergo a series of dysregulated reactions during the pathogenesis. The Sepsis guidelines^[1]^ indicate that serum lactic acid levels can reflect the changes in tissue perfusion; however, the serum lactic acid levels are not the only indicator reflect disorders in the metabolic parameters, reassessing the importance of metabolic disorders in patients with sepsis from a different perspective. Therefore, it is necessary to identify novel and representative markers of the metabolic dysfunction in sepsis. The anion gap (AG) is a comprehensive outcome indicator that is determined by the concentrations of the measured anions and cations,^[2]^ which reflects the acid–base metabolism, and has the potential to reflect the overall metabolic status of the patients. In the critical care clinical setting, the AG is used to differentiate between acid–base disorders and to determine the cause of metabolic acidosis.^[3]^ Moreover, the AG is a prognostic marker for other diseases,^[4,5]^ and shows a positive correlation with alcohol-related intoxication,^[6]^ early kidney disease,^[7]^ and advanced chronic kidney disease.^[8]^ Furthermore, the serum AG at admission is a predictor of mortality in the pediatric intensive care unit (ICU).^[9]^

Metabolic acidosis is usually associated with the severity of the condition of patients with sepsis or septic shock. AG, a parameter derived from blood chemistry, has been suggested as a prognostic indicator in sepsis. However, its association with short-term outcomes, such as 28-day mortality, in critically ill adult patients with sepsis, remains to be fully elucidated. This knowledge gap underscores the need for a comprehensive evaluation of the AG’s predictive value in this patient population. The primary objective of this retrospective cohort study is to investigate the association between the anion-gap and 28-day mortality in critically ill adult patients diagnosed with sepsis. By employing the PECO framework, we aim to define the population (P) of critically ill adults with sepsis, the exposure (E) of an elevated anion-gap, and the primary outcome (O) of interest, which is 28-day mortality. Additionally, we will explore secondary outcomes, such as 90-day mortality, length of stay (LOS)-ICU, length of hospital stays (LOS-hospital), the need for continuous renal replacement therapy (CRRT) and vasopressor use within the first 24 hours after admission. A high AG at admission is associated with an increased likelihood of in-hospital mortality.^[4]^ Thus, in this study, we aimed to determine the association between the initial serum AG and 28-day all-cause mortality in adult patients with sepsis. We hypothesized that, independent of baseline characteristics, a high AG at the time of ICU admission is associated with an increased risk of 28-day mortality in adult patients with sepsis.

## 2. Materials and methods

### 2.1. Study population

This retrospective cohort study screened 35,010 patients, who were selected based on the definitive Sepsis criteria.^[1]^ The main data for this study were obtained from the Medical Information Mart for Intensive Care IV (MIMIC-IV) database, The MIMIC-IV database is a comprehensive, de-identified clinical dataset originating from the Beth Israel Deaconess Medical Center in Boston, it includes extensive data on ICU patients, such as vital signs, lab results, and medication orders, spanning multiple years, accessible to researchers through an application process, MIMIC-IV supports critical care medicine research and development of clinical decision tools while adhering to strict ethical standards.^[10,11]^ MIMIC-IV database contains 4 modules (“core,” “hosp,” “icu,” and “ed”), and provided clinicodemographic patient data that were recorded from 2008 to 2019. One of the authors (Kang Zou) who passed the Training Initiative examination (certification number 39906164 by CITI program) obtained approval by PhysioNet to access the database. The findings of this study are reported in accordance with the Strengthening the Reporting of Observational Studies in Epidemiology (STROBE) reporting guidelines.^[12]^ In order to safeguard patient privacy, the data analyzed in this research did not include identifiable participant data; therefore, the need for informed consent was waived, and the Scientific Research Ethics Committee of the First Affiliated Hospital of Gannan Medical College approved this study (LLSC-2021083101).

### 2.2. Eligibility criteria

Adult patients (age ≥ 18 years) who met the Sepsis criteria were screened for inclusion. The exclusion criteria were as follows: (1) a history of ICU admission (a previous ICU admission in the same hospital admission); (2) a null value recorded for the AG; and (3) the presence of missing covariates and erroneous values. Referring to previous published articles using this database,^[13–15]^ the exclusion of patients with a history of ICU admissions ensures that each patient is an individual, as the MIMIC IV database is a single-center database.

### 2.3. Covariates

The following patient variables were examined: age, sex, ethnicity, length of ICU stay (LOS-ICU), LOS-hospital, and critical illness score, including the Simplified Acute Physiology Score II (SAPSII), Acute Physiology Score III (APSIII), Charlson Comorbidity Index, Oxford Acute Severity of Illness Score (OASIS), Glasgow Coma Scale score and SOFA_score. Other details that were obtained included the presence of comorbidities and chronic diseases, receipt of CRRT, and vasopressor use within the first 24 hours after admission. The vasopressors included dobutamine, dopamine, epinephrine, norepinephrine, phenylephrine, and vasopressin. Furthermore, we included the values of albumin, bicarbonate, blood urea nitrogen (BUN), calcium, creatinine, chloride, blood glucose concentrations; sodium, and potassium base excess; total CO_2_; serum lactate levels; pCO_2_; pH; pO_2_; and hematological parameters, such as the white blood cell count, international normalized ratio, prothrombin time, partial thromboplastin time, hemoglobin level, platelet count, red blood cell count, and red cell distribution width. We obtained the AG at baseline that we have chosen to measure the same time point as the other laboratory parameters, which were obtained from the first laboratory test on the first day of ICU admission, and which was recorded as a continuous variable (itemid = 50,868 from the laboratory events table of mimic_hosp), after deleting the missing data under the assumption of “missing at random.” We also have directly removed the patients with missing data of other variables. The code of data extraction is available on Github (https://github.com/MIT-LCP/mimic-iv).

### 2.4. Outcomes

The primary outcome was 28-day mortality. We identified the participants’ date of death using both admission records and the patients’ tables from mimic_core. Secondary outcomes included 90-day mortality, LOS-ICU, LOS-hospital, CRRT, and vasopressor use within the first 24 hours after admission.

### 2.5. Statistical analysis

We analyzed baseline characteristics of participants according to the principles of the serum AG-level quartiles. Continuous variables with normal distribution are expressed as mean ± SD, and data with skewed distribution are reported as the median (interquartile range); categorical variables are presented as frequencies or percentages.

A one-way analysis of variance for continuous variables with normality were applied to compare the characteristics of the study subjects among the AG quartile groups. Turkey method was used for multiple comparison. The Kruskal–Wallis test for data with skewed distribution. The chi-square test was used to compare patient characteristics between the 2 groups as appropriate for categorical variables. To determine the association between the early AG and 28-day mortality, an adjusted OR with a 95% confidence interval [95% CI] was calculated using logistic regression to account for potential confounders. Covariates influencing the OR by more than 10% were then included in the multivariable logistic regression models. We constructed the model to adjust for covariates and variables with multicollinearity were not included in the model adjustment. The variance inflation factor is a method for quantifying multicollinearity. If a variable has a variance inflation factor value >10, it indicates that the variable is highly collinear with other variables. The first model was adjusted for demographic variables, age, and sex; the second model additionally adjusted for LOS-hospital, SAPSII, APSIII, OASIS, CRRT, and SOFA_score. A final model further adjusted for medication and laboratory data variables, such as norepinephrine; vasopressin; lactate levels; albumin, bicarbonate, BUN, creatinine, and chloride concentrations. The analysis of 28-day mortality using the Kaplan–Meier curve and comparisons by the log-rank test were performed using the AG as the classification variable.

Smooth curve-fitting analysis with the AG as a variable was performed. As the serum AG is a continuous variable, we used generalized additive models to identify nonlinear relationships in order to better identify the actual relationships between exposures and outcomes. Furthermore, we conducted a sensitivity analysis to evaluate the robustness of the results. We then performed subgroup analysis, stratified by age, sex, ethnicity, sepsis classification, SOFA_score, and comorbidities, using the stratified logistic regression models. The modifications and interactions between subgroups were assessed using the likelihood-ratio tests. All analyses were performed using the statistical software package R 3.6.3 (http://www.R-project.org, The R Foundation) and Free Statistics software versions 1.5. A two-tailed test was performed, and *P* < .05 was considered statistically significant.

## 3. Results

### 3.1. Study population

**Figure 1.**
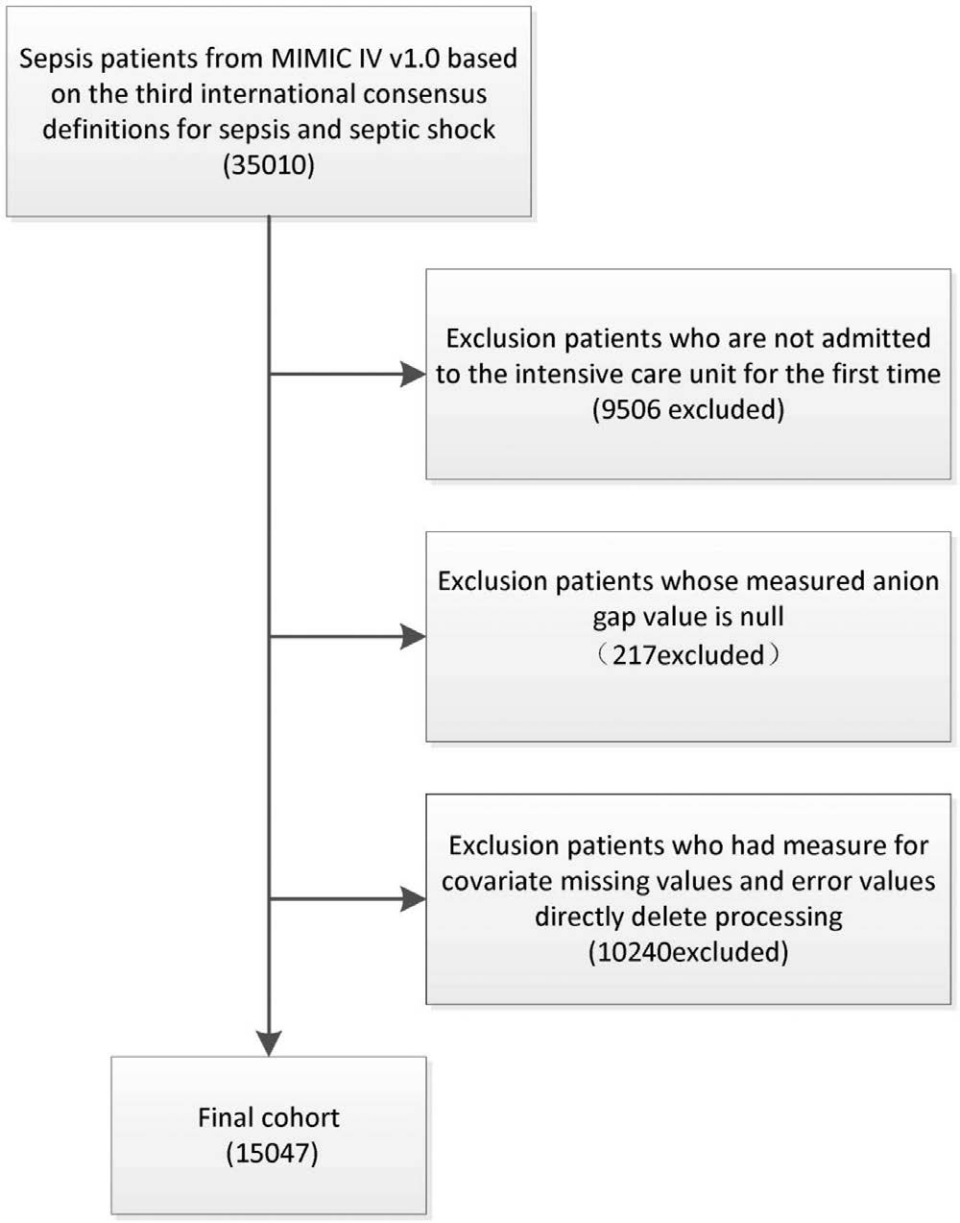
Flow diagram of the screening and enrollment of study participants.

The MIMIC-IV v1.0 database from the USA included data from 35,010 adult patients with sepsis, diagnosed based on the Sepsis definition. Following exclusion based on the abovementioned eligibility criteria, a total of 15,047 participants were finally recruited in this cohort. The enrollment process is illustrated in the flowchart shown in Figure 1.

### 3.2. Baseline characteristics

In this cohort study, baseline characteristics were grouped and displayed according to the AG quartiles of the exposure variable. In this study population of 15,047 participants (Table 1 and Table S1, Supplemental Digital Content, http://links.lww.com/MD/N248), the average age was 65.9 ± 16.0 years, and 6401 (42.5%) participants were female. Among these patients, 9945 (66.1%) were white, and 2686 (17.9%) died during the 28-day follow-up period. No statistically significant intergroup differences were observed for LOS-hospital and the presence of rheumatic diseases, peptic ulcer, and metastatic solid tumor among the different AG groups (all *P* > .05). Participants in the group with the highest AG (Q4) had the highest 28-day mortality and SAPSII, APSIII, OASIS scores, and SOFA_score compared to participants in the other groups. The highest AG (Q4) had the highest lactate levels, creatinine, glucose, the opposite pattern was observed the lowest for total CO_2_, pCO_2_, bicarbonate, and chloride levels than any of the other groups as show Figure S1, Supplemental Digital Content, http://links.lww.com/MD/N248. Different groups and different letters (abcd) representing a significant difference between the 2 groups, and different groups and the same letter representing a nonsignificant difference between the groups.

**Table 1:** Baseline characteristics and clinical laboratory parameters of the study populations.

Characteristics	Total participants	Serum anion gap quartile				*P* value
		Q1 (<12 mEq/L)	Q2 (12–15 mEq/L)	Q3 (15–18 mEq/L)	Q4 (≥18 mEq/L)	
Participants (n)	15,047	2900	4481	3835	3831	
Age, mean ± SD, year	65.9 ± 16.0	65.6 ± 15.2	66.0 ± 16.4	66.9 ± 16.0	65.1 ± 16.2	<.001
*Sex, n* (%)						.001
Female	6401 (42.5)	1147 (39.6)	1947 (43.5)	1691 (44.1)	1616 (42.2)	.001
Ethnicity, n (%)						<.001
White	9945 (66.1)	2022 (69.7)	3048 (68)	2562 (66.8)	2313 (60.4)	
Black/Asian/Hispanic	2596 (17.3)	447 (15.4)	728 (16.2)	662 (17.3)	759 (19.8)	
Unknown	2506 (16.7)	431 (14.9)	705 (15.7)	611 (15.9)	759 (19.8)	
*Primary and secondary outcomes*						
Death 28-day, n (%)	2686 (17.9)	321 (11.1)	589 (13.1)	688 (17.9)	1088 (28.4)	<.001
Death 90-day, n (%)	3119 (20.7)	385 (13.3)	709 (15.8)	800 (20.9)	1225 (32)	<.001
LOS ICU, median (IQR)	3.6 (1.9, 7.6)	3.2 (1.6, 6.8)	3.4 (1.8, 7.4)	3.7 (2.0, 8.0)	3.8 (2.0, 8.1)	<.001
LOS Hospital, Median (IQR)	9.7 (5.7, 17.0)	9.3 (5.7, 15.9)	9.7 (5.8, 16.9)	9.8 (5.9, 17.0)	9.8 (5.4, 17.9)	.216
*Critical illness score*						
SAPSII, median (IQR)	40.0 (31.0, 50.0)	37.0 (29.0, 45.0)	38.0 (30.0, 47.0)	40.0 (31.5, 49.0)	46.0 (36.0, 57.0)	<.001
APSIII, median (IQR)	55.0 (41.0, 75.0)	47.0 (35.0, 65.0)	50.0 (38.0, 69.0)	55.0 (41.0, 73.0)	67.0 (51.0, 91.0)	<.001
CCI, median (IQR)	5.0 (3.0, 7.0)	4.0 (3.0, 6.0)	5.0 (3.0, 6.0)	5.0 (3.0, 7.0)	5.0 (4.0, 7.0)	<.001
OASIS, median (IQR)	36.0 (29.0, 42.0)	34.0 (28.0, 41.0)	35.0 (29.0, 41.0)	35.0 (29.0, 42.0)	38.0 (31.0, 46.0)	<.001
GCS, median (IQR)	11.4 ± 4.0	11.6 ± 3.9	11.4 ± 4.0	11.4 ± 4.0	11.1 ± 4.2	.001
SOFA_score	3.0 (2.0, 4.0)	3.0 (2.0, 4.0)	3.0 (2.0, 4.0)	3.0 (2.0, 4.0)	4.0 (2.0, 5.0)	<.001
CRRT, n (%)	1478 (9.8)	87 (3)	217 (4.8)	316 (8.2)	858 (22.4)	<.001
Laboratory results	3.0 (2.0, 4.0)	3.0 (2.0, 4.0)	3.0 (2.0, 4.0)	3.0 (2.0, 4.0)	4.0 (2.0, 5.0)	<.001
Base excess, median (IQR), mEq/L	‐1.0 (‐5.0, 0.0)	0.0 (‐2.0, 2.0)	‐1.0 (‐3.0, 1.0)	‐1.0 (‐4.0, 0.0)	‐4.0 (‐9.0, ‐1.0)	<.001
TCO_2_, mean ± SD, mEq/L	23.7 ± 5.7	25.9 ± 5.4	24.7 ± 5.1	23.5 ± 5.1	20.9 ± 5.8	<.001
Lactate, median (IQR), mmol/L	1.5 (1.1, 2.4)	1.4 (1.0, 2.0)	1.4 (1.0, 2.1)	1.5 (1.1, 2.2)	1.9 (1.2, 3.8)	<.001
PCO_2_, median (IQR), mm Hg	39.0 (33.0, 45.0)	40.0 (35.0, 47.0)	39.0 (34.0, 45.0)	38.0 (33.0, 44.0)	37.0 (31.0, 44.0)	<.001
pH, mean ± SD, units	7.3 ± 0.1	7.4 ± 0.1	7.4 ± 0.1	7.3 ± 0.1	7.3 ± 0.1	<.001
WBC, median (IQR), K/uL	11.7 (8.6, 15.5)	11.2 (7.7, 14.3)	11.5 (8.3, 14.9)	11.7 (8.8, 15.6)	12.5 (9.8, 17.6)	<.001
Albumin, mean ± SD, g/dL	3.0 ± 0.7	3.0 ± 0.7	3.0 ± 0.7	3.0 ± 0.7	3.0 ± 0.7	.037
HCO_3_⁻, mean ± SD, mEq/L	21.1 ± 4.7	23.6 ± 4.7	22.3 ± 4.1	20.9 ± 4.0	18.2 ± 4.6	<.001
BUN, median (IQR), mg/dL	19.0 (13.0, 32.0)	16.0 (12.0, 22.0)	17.0 (13.0, 26.0)	20.0 (14.0, 32.0)	29.0 (17.0, 53.5)	<.001
Creatinine, median (IQR), mg/dL	1.0 (0.7, 1.5)	0.8 (0.6, 1.0)	0.9 (0.7, 1.2)	1.0 (0.7, 1.6)	1.5 (1.0, 2.8)	<.001
Chloride, mean ± SD, mEq/L	103.8 ± 6.4	105.9 ± 6.1	104.5 ± 5.6	103.5 ± 6.1	101.8 ± 7.2	<.001

Data presented are mean ± SD, median (IQR), or N (%).

APSIII = acute physiology score III; BUN = blood urea nitrogen; CCI = Charlson comorbidity index; CRRT = continuous blood purification treatment; GCS = Glasgow coma scale; IQR = Interquartile Range; LOS = length of stay; OASIS = oxford acute severity of illness score; SAPS II = simplified acute physiology score II; SD = standard deviation, WBC = white blood cell.

### 3.3. Primary outcome

In this study, we constructed 4 models for the logistic regression analysis of the independent effect of early AG on the 28-day mortality. The effect sizes (OR) and 95% CI are listed in Table 2. In the unadjusted model, the model-based effect size can be explained as the association between AG and 28-day mortality. For instance, an effect size of 1.56 for the 28-day mortality means that, in every increase of AG (per SD mEq/L), there is an associated 1.56 times (OR 1.56, 95% CI 1.5–1.62, *P* < .001) increase in the 28-day mortality. In the adjusted models I, II, and III, every increase in AG (per SD mEq/L) resulted in 1.59 times (OR 1.59, 95% CI 1.52–1.65, *P* < .001), 1.13 times (OR 1.13, 95% CI 1.07–1.19, *P* < .001), and 1.2 times (OR 1.2, 95% CI 1.12–1.29, *P* < .001) increase, respectively, in the 28-day mortality. Adjust I adjusts for age and sex, adjust II adjusts for adjust I plus LOS hospital, SAPSII, APSIII, OASIS, CRRT, SOFA_score, adjust III adjusts for adjust II plus norepinephrine, vasopressin, lactate, albumin, bicarbonate, bun, creatinine, chloride.

**Table 2:** Multivariable logistic regression analysis of the association between the anion-gap levels and 28-day mortality in patients with sepsis.

Variable	Total n	Event n%	Unadjusted		Adjusted I		Adjusted II		Adjusted III	
			OR (95 CI)	*P* value	OR (95 CI)	*P* value	OR (95 CI)	*P* value	OR (95 CI)	*P* value
*Serum anion gap continuous*										
Per SD (mEq/L) increment serum anion gap	15,047	2686 (17.9)	1.56 (1.5–1.62)	<.001	1.59 (1.52–1.65)	<.001	1.13 (1.07–1.19)	<.001	1.2 (1.12–1.29)	<.001
*Serum anion gap quintile*										
Q1 (<12 mEq/L)	2900	321 (11.1)	Ref.		Ref.		Ref.		Ref.	
Q2 (12-15 mEq/L)	4481	589 (13.1)	1.22 (1.05–1.41)	.008	1.21 (1.04–1.39)	.012	1.08 (0.92–1.26)	.365	1.16 (0.99–1.37)	.072
Q3 (15-18 mEq/L)	3835	688 (17.9)	1.76 (1.52–2.03)	<.001	1.72 (1.49–1.99)	<.001	1.33 (1.13–1.56)	.001	1.53 (1.29–1.81)	<.001
Q4 (≥18 mEq/L)	3831	1088 (28.4)	3.19 (2.78–3.65)	<.001	3.25 (2.84–3.72)	<.001	1.44 (1.23–1.67)	<.001	1.69 (1.4–2.04)	<.001
*P* for trend	15,047	2686 (17.9)	<.001		<.001		<.001		<.001	

Data presented are OR and 95% CI. Adjust I adjusts for age and sex, adjust II adjusts for adjust I plus LOS hospital, SAPSII, APSIII, OASIS, CRRT, SOFA_score, adjust III adjusts for adjust II plus norepinephrine, vasopressin, lactate, albumin, HCO_3_⁻, BUN, creatinine, chloride. Q1 (anion gap < 12mEq/L); Q2 (12 < anion gap ≤ 15 mEq/L); Q3 (15 < anion gap ≤ 18 mEq/L); Q4 (anion gap ≥ 18 mEq/L).

BUN = blood urea nitrogen.

For the sensitivity analysis, we converted AG from a continuous variable to a categorical variable, as the AG quartile Q1 (anion gap < 12mEq/L); Q2 (12 < anion gap ≤ 15 mEq/L); Q3 (15 < anion gap ≤ 18 mEq/L); Q4 (anion gap ≥ 18 mEq/L), and the *P*-value for the trend of the AG as a categorical variable in the adjusted model III was consistent with the result obtained with the AG as a continuous variable. Model I adjusts for age and sex, multiple logistic regression analysis revealed decreased 28-day mortality (OR 1.21, 95% CI 1.04–1.39, *P* = .012) in the Q2 and (OR 1.72, 95% CI 1.49–1.99, *P* < .001) in the Q3, and (OR 3.25, 95% CI 2.84–3.72, *P* < .001) in the Q4, Q1 as a reference group; Model II adjusts for age and sex, multiple logistic regression analysis revealed decreased 28-day mortality (OR 1.21, 95% CI 1.04–1.39, *P* = .012) in the Q2 and (OR 1.72, 95% CI 1.49–1.99, *P* < .001) in the Q3, and (OR 3.25, 95% CI 2.84–3.72, *P* < .001) in the Q4, Q1 as a reference group; Model II adjusts for model I plus LOS hospital, SAPSII, APSIII, OASIS, CRRT, SOFA_score, multiple logistic regression analysis revealed decreased 28-day mortality (OR 1.08, 95% CI 0.92–1.26, *P* = .365) in the Q2 and (OR 1.33, 95% CI 1.13–1.56, *P* < .001) in the Q3, and (OR 1.43, 95% CI 1.22–1.67, *P* < .001) in the Q4, Q1 as a reference group; Model III adjusts for model II plus norepinephrine, vasopressin, lactate, albumin, bicarbonate, bun, creatinine, chloride, multiple logistic regression analysis revealed decreased 28-day survival (OR 1.16, 95% CI 0.99–1.37, *P* = .072) in the Q2 and (OR 1.53, 95% CI 1.29–1.81, *P* < .001) in the Q3, and (OR 1.69, 95% CI 1.4–2.04, *P* < .001) in the Q4, Q1 as a reference group; as the AG increased, changes in the trends of the secondary outcomes, including the 90-day mortality (Table S3, Supplemental Digital Content, http://links.lww.com/MD/N248), LOS-ICU, LOS-hospital, CRRT, and vasopressor use within the first 24 hours after admission were consistent, as shown in Table 1 and Table S1, Supplemental Digital Content, http://links.lww.com/MD/N248.

### 3.4. Subgroup analysis

**Figure 2.**
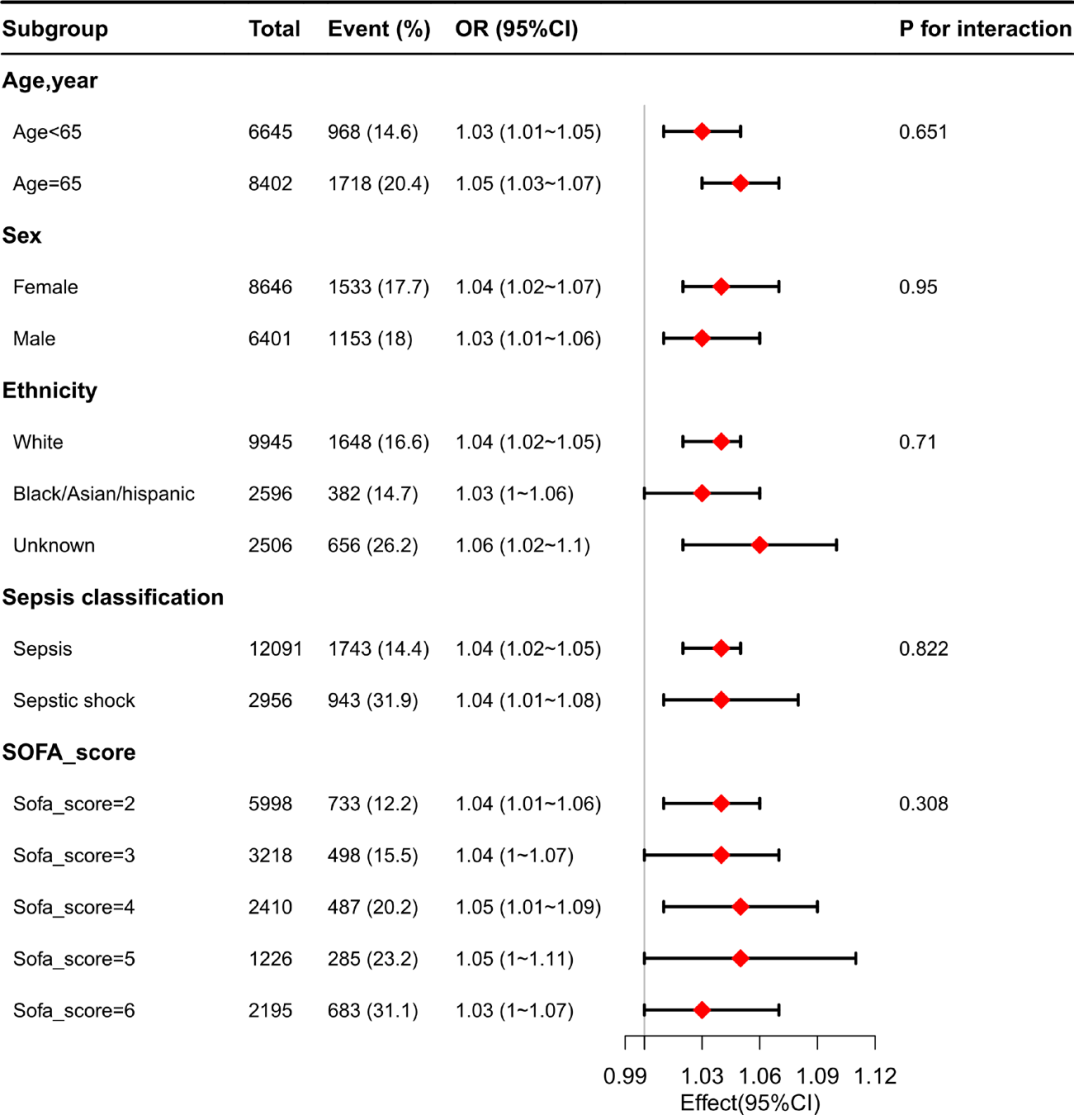
Subgroup analysis of the association between the anion-gap levels and 28-day mortality in patients with sepsis. By potential effect measure modifiers, Model adjusted for age, sex, LOS hospital, SAPSII, APSIII, OASIS, CRRT, SOFA_score, norepinephrine, vasopressin, lactate, albumin, bicarbonate, bun, creatinine, and chloride. Subgroup analysis in participants with sex, age, ethnicity, sepsis classification and SOFA_score showed that the *P* value for the interaction was more than .05.

The AG was significantly associated with the 28-day mortality in the sex-, age-, ethnicity-stratified sepsis classification and SOFA_score analyses, with the exception of black/Asian/Hispanic ethnicity (OR 1.03, 95% CI 1–1.06, *P* = .075), displayed in Figure 2. For the comorbidity-stratified patient subgroups of congestive heart failure, cerebrovascular disease, chronic pulmonary disease, and renal disease, the *P*-values were <.05. *P* for interaction of comorbidity subgroups were more than 0.05, except for malignant cancer (*P* = .007). The subgroup analysis was conducted after adjusting for age, sex, LOS-hospital, SAPSII, APSIII, OASIS, CRRT, SOFA_score, norepinephrine, vasopressin, lactate, albumin, bicarbonate, BUN, creatinine, and chloride levels (Fig. 2 and Table S2, Supplemental Digital Content, http://links.lww.com/MD/N248).

### 3.5. Positive association between the AG and 28-day mortality

**Figure 3.**
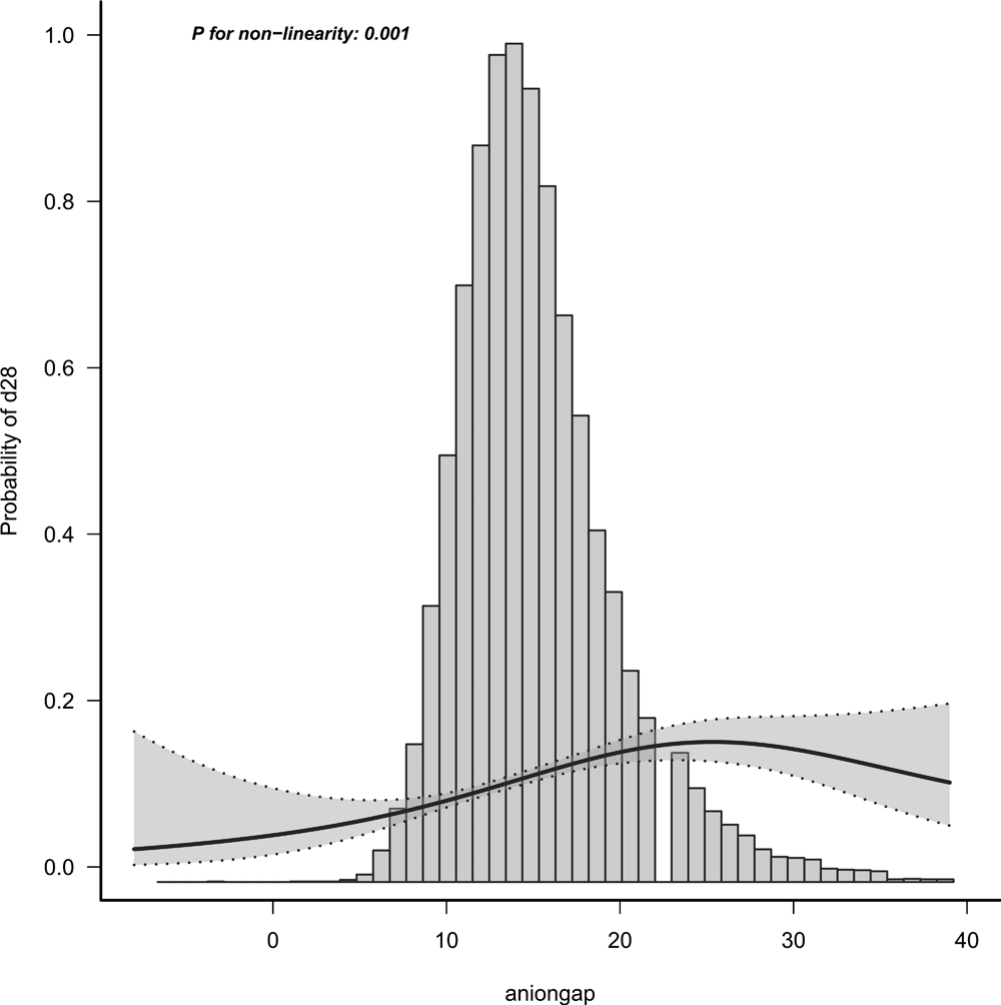
Smooth curve fitting of the relationship between serum anion gap levels and sepsis 28-day mortality. The positive relationship between the AG level and 28-day mortality was nearly nonlinear after adjusting for age, sex, LOS-hospital, SAPSII, APSIII, OASIS, CRRT, SOFA_score, norepinephrine, vasopressin, lactate, albumin, bicarbonate, BUN, creatinine, and chloride levels (*P* for nonlinearity = .001). BUN = blood urea nitrogen.

We analyzed the relationship between the AG and 28-day mortality, which showed a positive trend (Fig. 3). The smooth curve-fitting analysis and the result of the generalized additive model showed that the positive relationship between the AG level and 28-day mortality was nearly nonlinear after adjusting for age, sex, LOS-hospital, SAPSII, APSIII, OASIS, CRRT, SOFA_score, norepinephrine, vasopressin, and lactate, albumin, bicarbonate, BUN, creatinine, and chloride levels (*P* for nonlinearity = 0.001).

### 3.6. Positive association between the serum AG and lactate

We also analyzed the relationship between the serum AG and lactate, which showed a positive trend (Fig. S2, Supplemental Digital Content, http://links.lww.com/MD/N248). A linear relationship was found between the anion gap and the serum lactate line curve fit (*P* for linearity < .001).

### 3.7. Kaplan–Meier survival analysis

**Figure 4.**
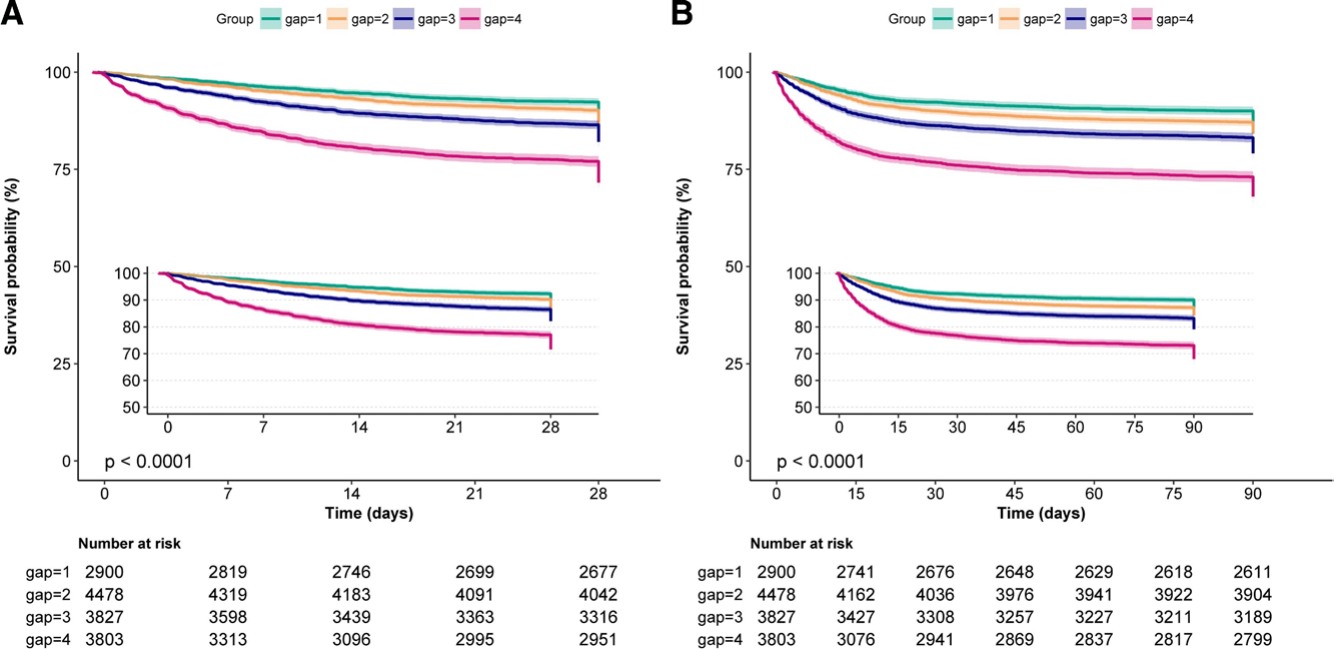
Kaplan–Meier survival curves of the 28-day mortality and 90-day mortality in sepsis patients with different serum anion gap levels.

The survival curve was used to demonstrate the 28-day all-cause mortality of sepsis in the serum AG-quartile groups. The survival probability was lower among patients with higher serum AG levels. Kaplan–Meier survival analysis showed that the highest AG-quartile group had the highest 28-day mortality and 90-day mortality compared with the other groups (Fig. 4).

## 4. Discussion

In this large cohort of ICU patients, a higher serum AG quartile was associated with an increased 28-day mortality in adult patients with sepsis. We adjusted for confounders through multiple regression analysis and determined the robustness of results through subgroup analyses. Furthermore, the analysis of Kaplan–Meier survival curves showed that the 28-day and 90-day mortality significantly increased with the increase in the quartiles of the serum AG levels.

Metabolic acidosis is usually associated with the severity of the condition of patients with sepsis. Lactate, Total serum carbon dioxide, serum anion gap (AG) are indicators of metabolic acidosis. Total serum carbon dioxide and lactate are strongly associated with sepsis prognosis.^[14,16]^ Our assessment of the association of AG with the prognosis of sepsis, as distinct from lactate and total serum carbon dioxide is a different perspective on the dangers of metabolic disorders in sepsis and is the significance of the study in this article, AG is also one of the indicators that are readily available clinically and have practical clinical guidance. We also uncover the relationship between serum lactate and AG, a linear relationship was found between the anion gap and the serum lactate line curve fit (*P* < .001). The AG is determined by the concentrations of unmeasured anions and cations, determined through blood biochemistry, and is calculated directly from the serum levels. The AG value directly reflects the body’s metabolism and is unaffected by respiratory function^[17]^; furthermore, the AG in specific ranges aids the differential diagnosis of the etiology and types of metabolic acidosis. Critically ill patients often develop hypoproteinemia, which can mask elevated AG levels.^[18]^ Therefore, calculating the albumin-corrected AG reduces the effect of low albumin on the AG and can facilitate the determination of the type of acidosis in critically ill patients.^[19]^ Recently, Jiang et al^[20]^ published the results of a similar study, using the same database, and reported the albumin-corrected AG, for predicting in-hospital mortality among ICU patients with sepsis using propensity score-matched analyses, as well as the AG that was directly calculated. However, in the abovementioned study, few emergency tests for the assessment of the albumin level were conducted immediately after ICU admission, and several patients did not undergo albumin tests within 24 hours of ICU admission. Albumin leakage in patients with sepsis during acute disease progression, along with early intensive therapeutic interventions (e.g., albumin therapy), prior to testing the albumin levels. A study that used data from the MIMIC-IV database showed that, in patients with sepsis, the presence of at least 920 patients who received combination therapy with albumin in the first 24 hours associated with increased 28-day mortality.^[21]^ Therefore, albumin-corrected AG values may not be suitable for studies, and data on the AG can be obtained by direct extraction in the MIMIC-IV database (itemid = 50,868 from the laboratory events table of mimic_hosp). We directly extracted AG the first test value of patients admitted to the ICU for the first time to evaluate the association of the AG with the 28-day mortality and strictly followed the approach of the cohort study analysis by performing grouped analysis with the AG as the exposure factor to generate the results. We believe that the nonuse of the albumin-corrected AG facilitates the identification of a meaningful relationship between the AG at the time of ICU admission and the 28-day mortality. Thus, if the use of the albumin-corrected AG clarifies and strengthens the relationship between the AG level at the time of ICU admission and the 28-day mortality. The result of the generalized additive model showed that the positive relationship between an early AG and 28-day mortality was nearly nonlinear after adjusting for additional potential confounders in adult patients with sepsis.

Although this large cohort study was designed to explore the relationship between the serum AG and the clinical outcome in adult patients with sepsis, the study has some limitations. First, as the study was based on a publicly accessible database, there may be concerns regarding the generalizability of the conclusions; moreover, there may be confounding bias due to missing data. However, this database has been utilized by several authors worldwide and the resulting publications guarantee the data quality.^[21–24]^ Second, we only included patients with sepsis for whom the AG measurements were available, which could cause selection bias as the missing data were assumed to be deleted for random reasons. Third, we assessed only the initial AG, and the repeated assessment of changes in the AG may have provided more information. Fourth, we did not use the albumin-corrected AG and, instead, tested this hypothesis differently. After adjusting for covariates, we found a positive relationship between early serum AG and 28-day mortality in adult patients with sepsis. Fifth, The history of ICU admission refers to previous ICU stays at the same hospital, while data on previous ICU admissions at any hospital are not available in the MIMIC database. Six, as this was a retrospective cohort study, the analysis is susceptible to potential confounding factors. We cannot rule out the possibility of unmeasured confounders, although we used strict statistical adjustments to minimize the influence of potential confounders. We generated an E-value to assess the unmeasured confounding was 1.49. The primary findings were robust, unless an odds ratio for unmeasured confounders would need to be at least 1.49 between exposure and outcome (Fig. S3, Supplemental Digital Content, http://links.lww.com/MD/N248). Seventh, the limitations of existing studies in adjusting for confounding factors and not considering interactions or nonlinear relationships between covariates and outcomes. The complexity of the model was not added for good interpretation of the results. Finally, as with most single-center studies, our research subjects were Americans, and this detracts from the generalizability of the results, which may lack external validity for other ethnic populations. The 28-day all-cause mortality was evaluated in this study; however, we did not explore the causes of death in the study population. Therefore, the strength of the correlation of AG may vary for different causes. The data available in the MIMIC-IV database could not facilitate a competing-risk analysis. Therefore, the relationship between the AG and the 28-day mortality in patients with sepsis needs to be further explored in a more rigorous prospective study.

## 5. Conclusions

In adult patients with sepsis, a high AG at the time of ICU admission may be associated with a decreased possibility of survival at 28 days. We found a positive correlation between the AG and the 28-day mortality. The early AG at the time of ICU admission is an independent risk factor for prognosis.

## Acknowledgments

We are grateful to MIMIC for providing official data support, and to Liu Jie and his team at Beijing 301 Vascular Surgery Department and Free Statistics for technical support.

## Author contributions

**Conceptualization:** Zeying Lou, Huasheng Zhou.

**Data curation:** Zeying Lou.

**Funding acquisition:** Kang Zou.

**Formal analysis:** Fanghua Zeng.

**Methodology:** Li Xiao, Kang Zou.

**Project administration:** Kang Zou, Huasheng Zhou.

**Software:** Wenbao Huang, Li Xiao, Kang Zou.

**Supervision:** Wenbao Huang, Li Xiao, Kang Zou.

**Validation:** Kang Zou.

**Visualization:** Huasheng Zhou.

**Writing – original draft:** Zeying Lou, Kang Zou, Huasheng Zhou.

**Writing – review & editing:** Li Xiao, Kang Zou, Huasheng Zhou.

## Supplementary Material

## References

[1] Singer M Deutschman CS Seymour CW. The Third international consensus definitions for sepsis and septic shock (Sepsis-3). JAMA. 2016;315:801–10. 26903338 10.1001/jama.2016.0287 PMC4968574

[2] Oh MS Carroll HJ. The anion gap. N Engl J Med. 1977;297:814–7. 895822 10.1056/NEJM197710132971507

[3] Reilly RF Anderson RJ. Interpreting the anion gap. Crit Care Med. 1998;26:1771–2. 9824056 10.1097/00003246-199811000-00003

[4] Domínguez-Cherit G Namendys-Silva SA. Changes in the anion gap: a novel marker of outcome in critically ill patients. Back to the basis. Crit Care Med. 2013;41:336–7. 23269138 10.1097/CCM.0b013e318270e799

[5] Mohr NM Vakkalanka JP Faine BA. Serum anion gap predicts lactate poorly, but may be used to identify sepsis patients at risk for death: a cohort study. J Crit Care. 2018;44:223–8. 29175046 10.1016/j.jcrc.2017.10.043

[6] Kraut JA Kurtz I. Toxic alcohol ingestions: clinical features, diagnosis, and management. Clin J Am Soc Nephrol. 2008;3:208–25. 18045860 10.2215/CJN.03220807

[7] Abramowitz MK Hostetter TH Melamed ML. The serum anion gap is altered in early kidney disease and associates with mortality. Kidney Int. 2012;82:701–9. 22622500 10.1038/ki.2012.196 PMC3434284

[8] Asahina Y Sakaguchi Y Kajimoto S. Association of time-updated anion gap with risk of kidney failure in advanced CKD: a cohort study. Am J Kidney Dis. 2022;79:374–82. 34280508 10.1053/j.ajkd.2021.05.022

[9] Kim MJ Kim YH Sol IS. Serum anion gap at admission as a predictor of mortality in the pediatric intensive care unit. Sci Rep. 2017;7:1456. 28469150 10.1038/s41598-017-01681-9 PMC5431089

[10] Johnson AEW Bulgarelli L Shen L. MIMIC-IV, a freely accessible electronic health record dataset. Sci Data. 2023;10:1. 36596836 10.1038/s41597-022-01899-x PMC9810617

[11] Goldberger A Amaral L Glass L. PhysioBank, PhysioToolkit, and PhysioNet: components of a new research resource for complex physiologic signals. Circulation 2000;101:e215–20. 10851218 10.1161/01.cir.101.23.e215

[12] von Elm E Altman DG Egger M. Strengthening the Reporting of Observational Studies in Epidemiology (STROBE) statement: guidelines for reporting observational studies. BMJ. 2007;335:806–8. 17947786 10.1136/bmj.39335.541782.AD PMC2034723

[13] Chen H Zhu Z Zhao C. Central venous pressure measurement is associated with improved outcomes in septic patients: an analysis of the MIMIC-III database. Crit Care. 2020;24:433. 32665010 10.1186/s13054-020-03109-9 PMC7358999

[14] Chen H Zhao C Wei Y Jin J. Early lactate measurement is associated with better outcomes in septic patients with an elevated serum lactate level. Crit Care. 2019;23:351. 31711512 10.1186/s13054-019-2625-0 PMC6849274

[15] Feng M McSparron JI Kien DT. Transthoracic echocardiography and mortality in sepsis: analysis of the MIMIC-III database. Intensive Care Med. 2018;44:884–92. 29806057 10.1007/s00134-018-5208-7

[16] Kim JH Jang DH Jo YH. Serum total carbon dioxide as a prognostic factor for 28-day mortality in patients with sepsis. Am J Emerg Med. 2021;44:277–83. 32303411 10.1016/j.ajem.2020.04.006

[17] He X Liao X Xie Z Jiang C Kang Y. [Albumin corrected anion gap is an independent risk factor for long-term mortality of patients with sepsis]. Zhonghua Wei Zhong Bing Ji Jiu Yi Xue. 2017;29:117–21. 28625257 10.3760/cma.j.issn.2095-4352.2017.02.005

[18] Nanji AA Campbell DJ Pudek MR. Decreased anion gap associated with hypoalbuminemia and polyclonal gammopathy. JAMA. 1981;246:859–60. 6166764

[19] Hatherill M Waggie Z Purves L Reynolds L Argent A. Correction of the anion gap for albumin in order to detect occult tissue anions in shock. Arch Dis Child. 2002;87:526–9. 12456555 10.1136/adc.87.6.526 PMC1755806

[20] Hu T Zhang Z Jiang Y. Albumin corrected anion gap for predicting in-hospital mortality among intensive care patients with sepsis: a retrospective propensity score matching analysis. Clin Chim Acta. 2021;521:272–7. 34303712 10.1016/j.cca.2021.07.021

[21] Zhou S Zeng Z Wei H Sha T An S. Early combination of albumin with crystalloids administration might be beneficial for the survival of septic patients: a retrospective analysis from MIMIC-IV database. Ann Intensive Care. 2021;11:42. 33689042 10.1186/s13613-021-00830-8 PMC7947075

[22] Liu T Zhao Q Du B. Effects of high-flow oxygen therapy on patients with hypoxemia after extubation and predictors of reintubation: a retrospective study based on the MIMIC-IV database. BMC Pulm Med. 2021;21:160. 33985472 10.1186/s12890-021-01526-2 PMC8118109

[23] Zhao QY Liu LP Luo JC. A machine-learning approach for dynamic prediction of sepsis-induced coagulopathy in critically Ill patients with sepsis. Front Med (Lausanne). 2020;7:637434. 33553224 10.3389/fmed.2020.637434 PMC7859637

[24] Zhao QY Wang H Luo JC. Development and validation of a machine-learning model for prediction of extubation failure in intensive care units. Front Med (Lausanne). 2021;8:676343. 34079812 10.3389/fmed.2021.676343 PMC8165178

