# Supplementary material for: Association between the anion-gap and 28-day mortality in critically ill adult patients with sepsis: A retrospective cohort study
Source: Medicine (Baltimore). 2024 Jul 26;103(30):e39029. doi: 10.1097/MD.0000000000039029 (PMC11272324; doi:10.1097/MD.0000000000039029)

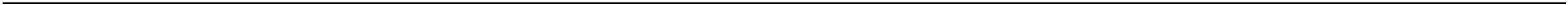

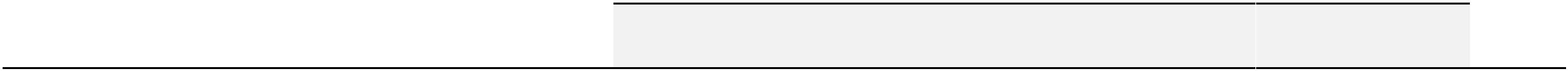

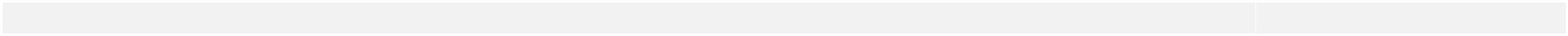

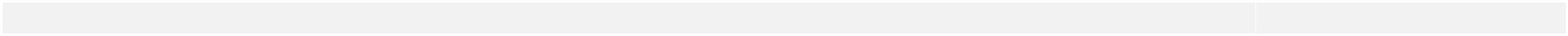

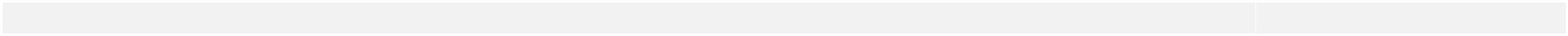

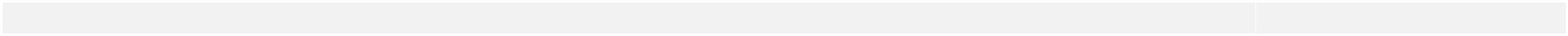

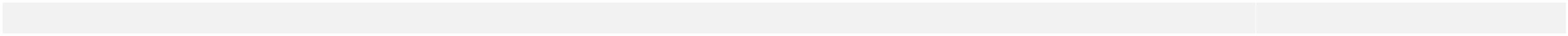

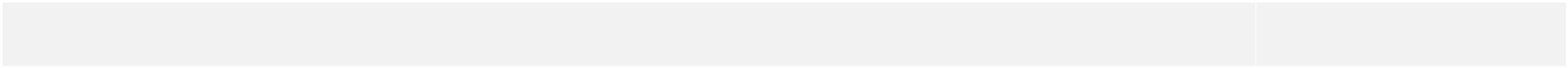

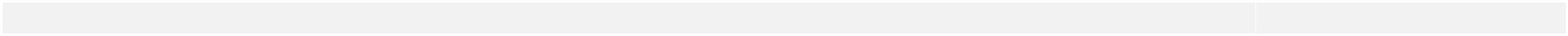

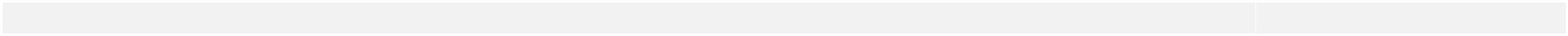

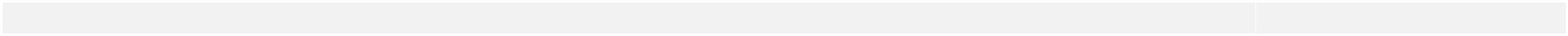

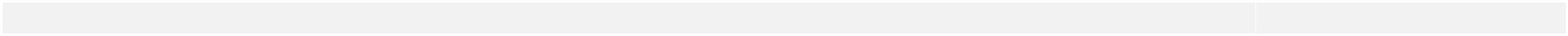

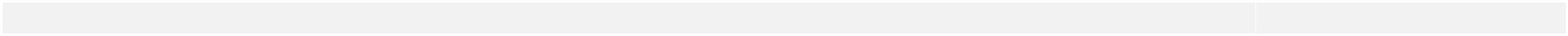

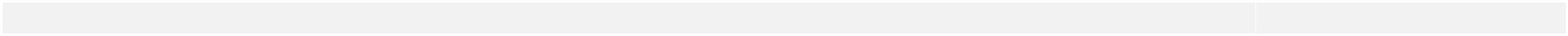

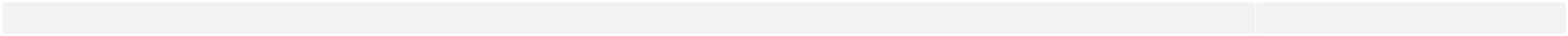

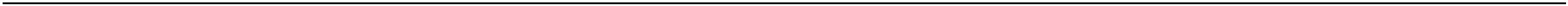
**Supplementary table 1. Continued baseline characteristics and clinical laboratory parameters of the study population.**

|  | **Characteristics** |  | **Total participants** |  | **Serum anion gap quartile**  **Q1 (＜12 mEq/L) Q2 (12-15 mEq/L) Q3 (15-18 mEq/L) Q4 (≥18 mEq/L)** |  | ***P***  **value** |
| --- | --- | --- | --- | --- | --- | --- | --- |

**Participants (n)** 15047 2900 4481 3835 3831 **Comorbidity**

**Myocardial Infarct, n (%)** 1397 (9.3) 243 (8.4) 367 (8.2) 366 (9.5) 421 (11) < 0.001
**Congestive Heart Failure, n (%)** 2952 (19.6) 470 (16.2) 828 (18.5) 784 (20.4) 870 (22.7) < 0.001
**Peripheral Vascular Disease, n (%)** 1068 (7.1) 240 (8.3) 257 (5.7) 265 (6.9) 306 (8) < 0.001
**Cerebrovascular Disease, n (%)** 1307 (8.7) 233 (8) 377 (8.4) 385 (10) 312 (8.1) 0.007
**Dementia, n (%)** 375 (2.5) 60 (2.1) 102 (2.3) 119 (3.1) 94 (2.5) 0.031
**Chronic pulmonary disease, n (%)** 2980 (19.8) 634 (21.9) 952 (21.2) 700 (18.3) 694 (18.1) < 0.001
**Rheumatic disease, n (%)** 427 (2.8) 73 (2.5) 135 (3) 104 (2.7) 115 (3) 0.537
**Peptic ulcer disease, n (%)** 263 (1.7) 44 (1.5) 83 (1.9) 65 (1.7) 71 (1.9) 0.684
**Mild liver disease, n (%)** 1947 (12.9) 328 (11.3) 455 (10.2) 467 (12.2) 697 (18.2) < 0.001
**Diabetes without complication, n** 3018 (20.1) 514 (17.7) 838 (18.7) 812 (21.2) 854 (22.3) < 0.001
**(%)**

**Diabetes with complication, n (%)** 1431 (9.5) 194 (6.7) 322 (7.2) 376 (9.8) 539 (14.1) < 0.001
**Paraplegia, n (%)** 452 (3.0) 90 (3.1) 128 (2.9) 149 (3.9) 85 (2.2) < 0.001
**Renal disease, n (%)** 2793 (18.6) 286 (9.9) 665 (14.8) 769 (20.1) 1073 (28) < 0.001
**Malignant cancer, n (%)** 1634 (10.9) 266 (9.2) 507 (11.3) 456 (11.9) 405 (10.6) 0.003
**Severe liver disease, n (%)** 880 (5.8) 148 (5.1) 195 (4.4) 188 (4.9) 349 (9.1) < 0.001
**Metastatic solid tumor, n (%)** 733 (4.9) 123 (4.2) 221 (4.9) 203 (5.3) 186 (4.9) 0.262
**Vasopressors**

**Dobutamine, n (%)** 257 (1.7) 15 (0.5) 37 (0.8) 56 (1.5) 149 (3.9) < 0.001
**Dopamine, n (%)** 536 (3.6) 61 (2.1) 123 (2.7) 139 (3.6) 213 (5.6) < 0.001
**Epinephrine, n (%)** 830 (5.5) 178 (6.1) 188 (4.2) 161 (4.2) 303 (7.9) < 0.001
**Norepinephrine, n (%)** 4188 (27.8) 612 (21.1) 995 (22.2) 1018 (26.5) 1563 (40.8) < 0.001
**Phenylephrine, n (%)** 2917 (19.4) 773 (26.7) 837 (18.7) 597 (15.6) 710 (18.5) < 0.001
**Vasopressin, n (%)** 1215 (8.1) 118 (4.1) 230 (5.1) 275 (7.2) 592 (15.5) < 0.001
**Neuroblock, n (%)** 486 (3.2) 65 (2.2) 113 (2.5) 106 (2.8) 202 (5.3) < 0.001
**Laboratory results**


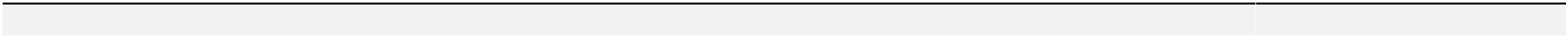

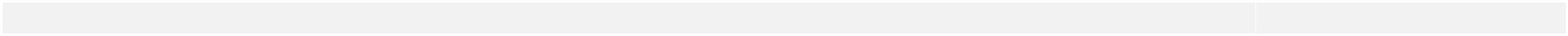

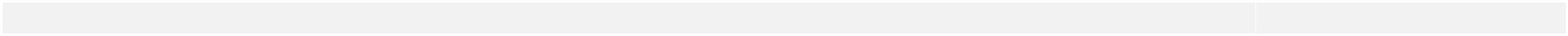

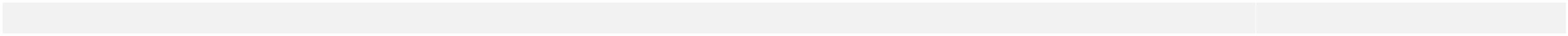

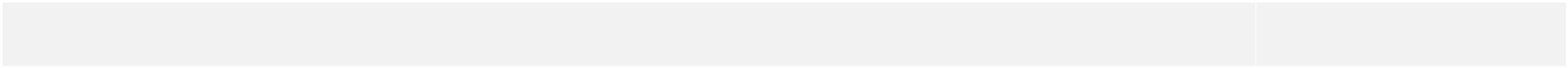

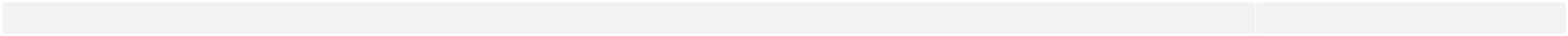

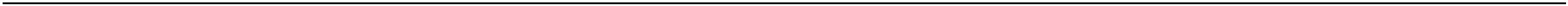
**Po_2_, Median (IQR), mmHg** 111.0 (58.0, 199.0) 138.0 (74.0, 275.2) 118.0 (65.0, 209.0) 106.0 (56.0, 177.0) 94.0 (47.0, 164.0) < 0.001
**Calcium, Mean ± SD, mg/dL** 8.1 ± 1.0 7.9 ± 1.0 8.1 ± 1.0 8.1 ± 1.0 8.1 ± 1.2 < 0.001
**Sodium, Mean ± SD, mEq/L** 136.9 ± 5.8 137.2 ± 5.5 137.1 ± 5.4 136.9 ± 5.7 136.4 ± 6.6 < 0.001
**Potassium, Mean ± SD, mEq/L** 4.0 ± 0.7 3.9 ± 0.6 3.9 ± 0.6 4.0 ± 0.7 4.2 ± 0.9 < 0.001
**Hemoglobin, Mean ± SD, g/dL** 10.4 ± 2.2 10.1 ± 2.0 10.5 ± 2.1 10.6 ± 2.2 10.5 ± 2.4 < 0.001

|  | **Platelet, Median (IQR), K/uL** 169.0 (122.0,  232.0) |  | 157.0 (118.0,  215.0) |  | 174.0 (126.0,  233.0) |  | 174.0 (125.0,  238.0) |  | 167.0 (117.0,  238.0) |  | < 0.001 |
| --- | --- | --- | --- | --- | --- | --- | --- | --- | --- | --- | --- |

**RBC, Mean ± SD, m/uL** 3.3 ± 0.8 3.2 ± 0.7 3.3 ± 0.7 3.3 ± 0.8 3.3 ± 0.9 < 0.001
**RDW, Mean ± SD** 15.3 ± 2.4 15.1 ± 2.3 15.1 ± 2.1 15.3 ± 2.4 15.7 ± 2.6 < 0.001
**Glucose, Median (IQR), mg/dL** 124.0 (107.0, 117.0 (104.0, 121.0 (106.0, 127.0 (108.0, 135.0 (108.0, < 0.001
 157.0) 140.0) 147.0) 161.0) 185.5)

**INR, Median (IQR)** 1.2 (1.1, 1.5) 1.2 (1.1, 1.4) 1.2 (1.1, 1.4) 1.2 (1.1, 1.5) 1.3 (1.1, 1.7) < 0.001
 **PT, Median (IQR), sec** 13.8 (12.3, 16.2) 14.0 (12.4, 15.9) 13.6 (12.2, 15.7) 13.6 (12.2, 15.9) 14.2 (12.3, 18.2) < 0.001
 **PTT, Median (IQR), sec** 30.1 (26.5, 36.3) 29.9 (26.5, 34.8) 29.6 (26.3, 35.0) 29.7 (26.3, 35.6) 31.3 (27.0, 41.2) < 0.001
Supplementary table 1. Baseline characteristics and clinical laboratory parameters of the study population. Data presented are mean±SD, median (IQR), or N (%).
Abbreviations: RDW, Red blood Cell distribution width; INR, International normalized ratio; PT, Prothrombin time; PTT, Partial thromboplastin time; IQR,
Interquartile Range; SD, Standard Deviation.


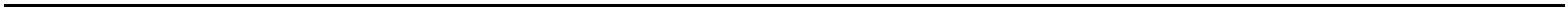

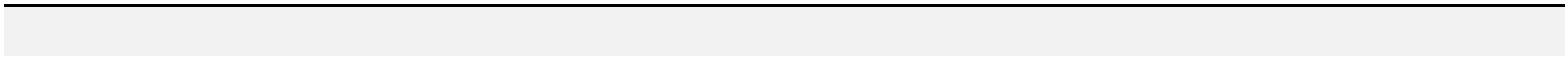

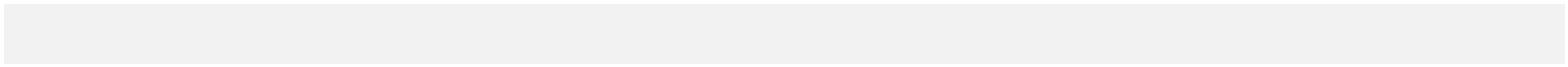

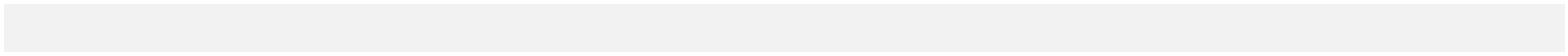

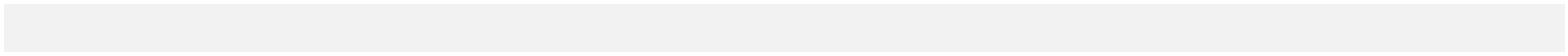

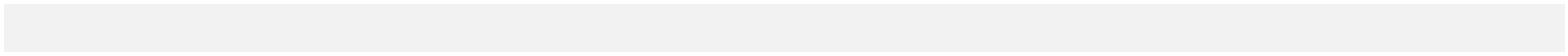

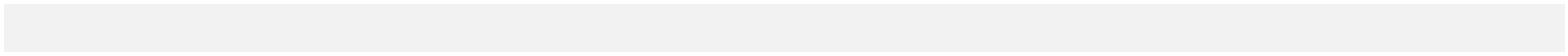

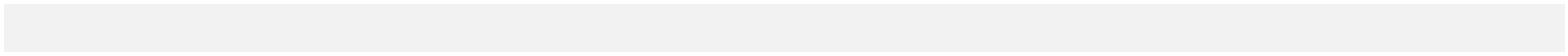

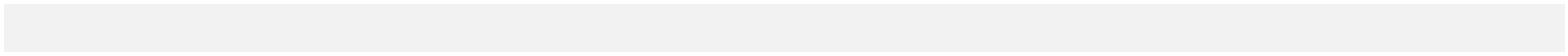

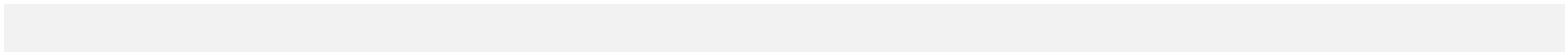

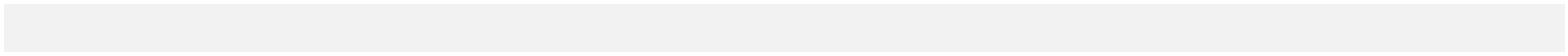

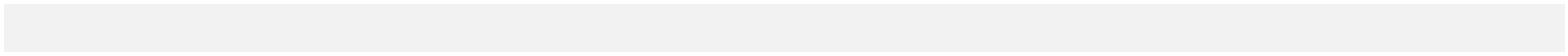

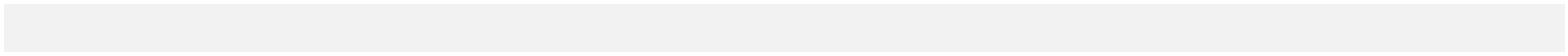

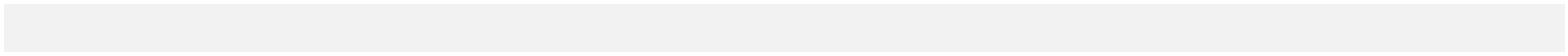

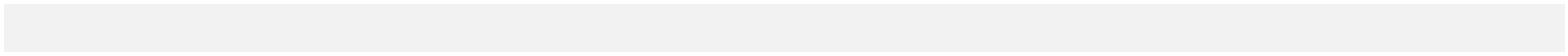

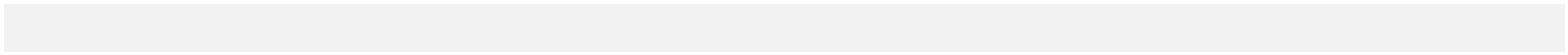

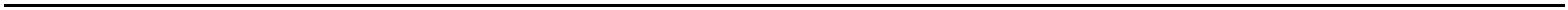
**Supplementary table 2：Subgroup analysis in participants with comorbidity.**

**Subgroup Total n Event n% OR (95%CI) *P* value *P* for interaction**

**Comorbidity Myocardial infarct**

**No** 13650 2330 (17.1) 1.04 (1.02~1.06) <0.001 0.554

**Yes** 1397 356 (25.5) 1.04 (1~1.09) 0.067 **Congestive heart failure**

**No** 12095 2004 (16.6) 1.04 (1.02~1.06) <0.001 0.789
 **Yes** 2952 682 (23.1) 1.05 (1.02~1.08) 0.001
**Peripheral vascular disease**

**No** 13979 2406 (17.2) 1.04 (1.03~1.06) <0.001 0.961
 **Yes** 1068 280 (26.2) 1.05 (1~1.1) 0.059
**Cerebrovascular disease**

**No** 13740 2381 (17.3) 1.04 (1.02~1.06) <0.001 0.308
**Yes** 1307 305 (23.3) 1.07 (1.03~1.12) 0.002

**Dementia**

**No** 14672 2617 (17.8) 1.04 (1.03~1.06) <0.001 0.486
 **Yes** 375 69 (18.4) 1.04 (0.94~1.15) 0.401
**Chronic pulmonary disease**

**No** 12067 2041 (16.9) 1.04 (1.02~1.05) <0.001 0.669
**Yes** 2980 645 (21.6) 1.06 (1.03~1.09) <0.001

**Rheumatic disease**

**No** 14620 2587 (17.7) 1.05 (1.03~1.06) <0.001 0.21
**Yes** 427 99 (23.2) 0.98 (0.89~1.07) 0.588

**Peptic ulcer disease**

**No** 14784 2626 (17.8) 1.04 (1.03~1.06) <0.001 0.264
**Yes** 263 60 (22.8) 1.01 (0.9~1.13) 0.917

**Mild liver disease**

**No** 13100 2136 (16.3) 1.06 (1.04~1.07) <0.001 0.131
**Yes** 1947 550 (28.2) 0.99 (0.95~1.02) 0.489

**Diabetes without cc**

**No** 12029 2123 (17.6) 1.05 (1.03~1.07) <0.001 0.843


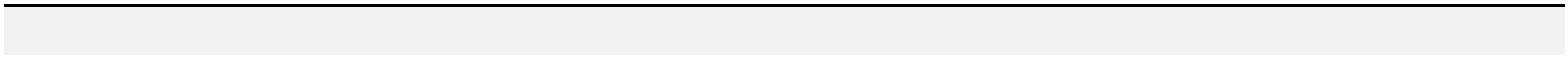

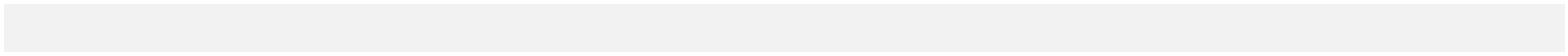

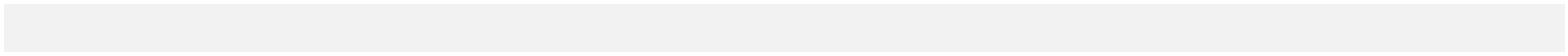

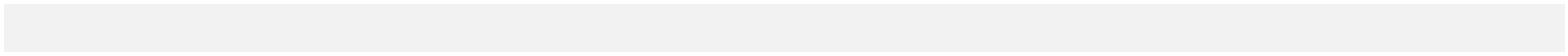

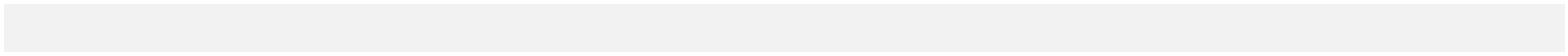

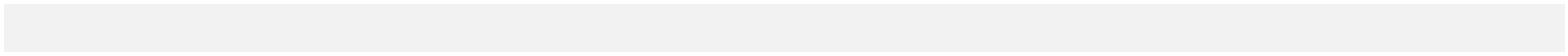

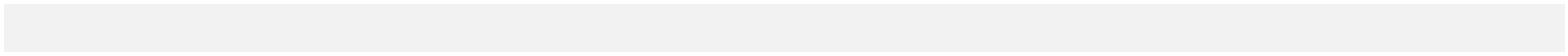

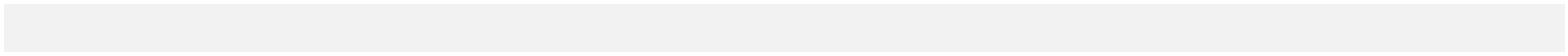

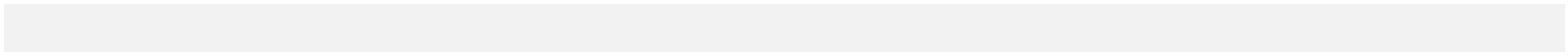

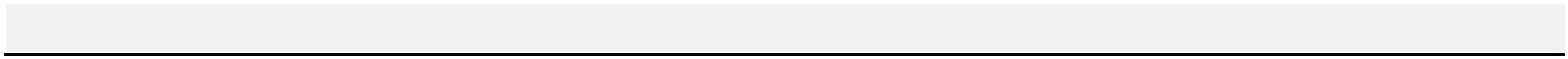
**Yes** 3018 563 (18.7) 1.03 (1~1.06) 0.052 **Diabetes with cc**

**No** 13616 2439 (17.9) 1.05 (1.03~1.06) <0.001 0.195
**Yes** 1431 247 (17.3) 1.01 (0.98~1.05) 0.472

**Paraplegia**

**No** 14595 2592 (17.8) 1.04 (1.03~1.06) <0.001 0.7
**Yes** 452 94 (20.8) 1.06 (0.97~1.17) 0.212

**Renal disease**

**No** 12254 2104 (17.2) 1.04 (1.03~1.06) <0.001 0.335
**Yes** 2793 582 (20.8) 1.05 (1.02~1.08) 0.003

**Malignant cancer**

**No** 13413 2264 (16.9) 1.05 (1.03~1.06) <0.001 0.007
**Yes** 1634 422 (25.8) 1.02 (0.97~1.06) 0.475

**Severe liver disease**

**No** 14167 2389 (16.9) 1.05 (1.04~1.07) <0.001 0.18
**Yes** 880 297 (33.8) 1 (0.95~1.05) 0.877

**Metastatic solid tumor**

**No** 14314 2441 (17.1) 1.04 (1.03~1.06) <0.001 0.357 **Yes** 733 245 (33.4) 1.03 (0.97~1.1) 0.389

Supplementary table 2: Subgroup analysis in participants with comorbidity. By potential effect measure modifiers, Model adjusted for age, sex, LOS hospital,
SAPSII, APSIII, OASIS, CRRT, SOFA_score, norepinephrine, vasopressin, lactate, albumin, bicarbonate, bun, creatinine, chloride. Subgroup analysis in
participants with comorbidity showed that the *P* value for the interaction was more than 0.05, however except malignant cancer.


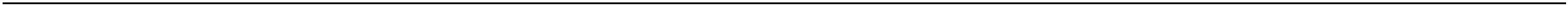

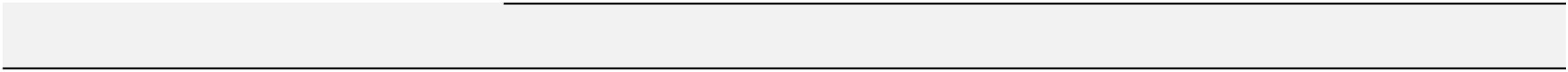

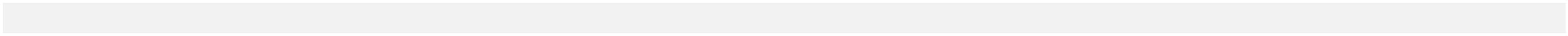

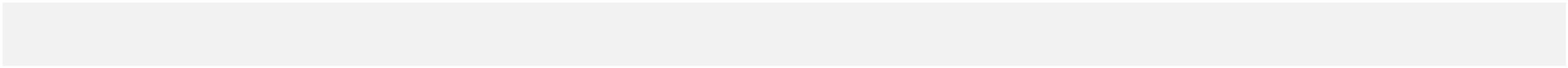

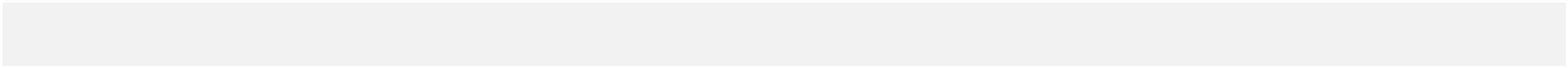

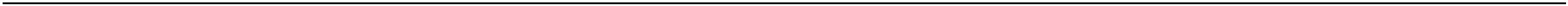
**Supplementary table 3： Multivariable logistic regression analysis of the association between the anion-gap levels and 90-day mortality in patients with**

**sepsis.**

**Unadjusted Adjusted I Adjusted II Adjusted III
Variable** Total Event n% OR (95CI) *P* OR (95CI) *P* OR (95CI) *P* OR (95CI) *P* n value value value value
**per SD (mEq/L)** 15047 3119 1.54 (1.48~1.6) <0.00 1.57 <0.001 1.16 <0.001 1.16 (1.1~1.24) <0.001
**increment serum anion** (20.7) 1 (1.51~1.63) (1.11~1.22)
**gap**

**Serum anion gap quintile**

2900 385 (13.3) Ref. Ref. Ref. Ref.

**Q1 (＜12 mEq/L)**

**Q2 (12-15 mEq/L)** 4481 709 (15.8) 1.23 (1.07~1.4) 0.003 1.22 0.004 1.11 0.153 1.16 (1~1.35) 0.045

(1.04~1.39) (0.96~1.28)

|  | **Q3 (15-18 mEq/L)** 3835 800 (20.9) 1.72  (1.51~1.97) |  | <0.00  1 |  | 1.69  (1.48~1.93) |  | <0.001 1.34  (1.16~1.55) |  | 0.001 1.45  (1.24~1.68) |  | <0.001 |
| --- | --- | --- | --- | --- | --- | --- | --- | --- | --- | --- | --- |

**Q4 (≥18 mEq/L)** 3831 1225 (32) 3.07 (2.7~3.49) <0.00 3.13 <0.001 1.54 <0.001 1.6 (1.35~1.9) <0.001

1 (2.76~3.56) (1.33~1.78)

|  | ***P* for the trend** 15047 3119  (20.7) |  | <0.001 <0.001 <0.001 <0.001 |
| --- | --- | --- | --- |

Supplementary table 3： Multivariable logistic regression analysis of the association between the anion-gap levels and 90-day mortality in patients with sepsis.

Data presented are OR and 95% CI. Adjust I adjusts for age and sex, adjust II adjusts for adjust I plus SAPSII, APSIII, OASIS, CRRT, SOFA_score, adjust III

adjusts for adjust II plus norepinephrine, vasopressin, lactate, albumin, bicarbonate, bun, creatinine, chloride. Q1 (anion gap＜12mEq/L); Q2 (12＜anion gap≤15

mEq/L); Q3 (15＜anion gap≤18 mEq/L); Q4 (anion gap≥18 mEq/L).

**Supplementary figure 1.** The results of the post-hoc analysis. A statistically significant difference was found between the four groups through the ANOVA test. Supplementary figure 1 to show the difference between two groups. Different groups and different letters (abcd) representing a significant difference between the two groups, and different groups and the same letter representing a non-significant difference between the groups.


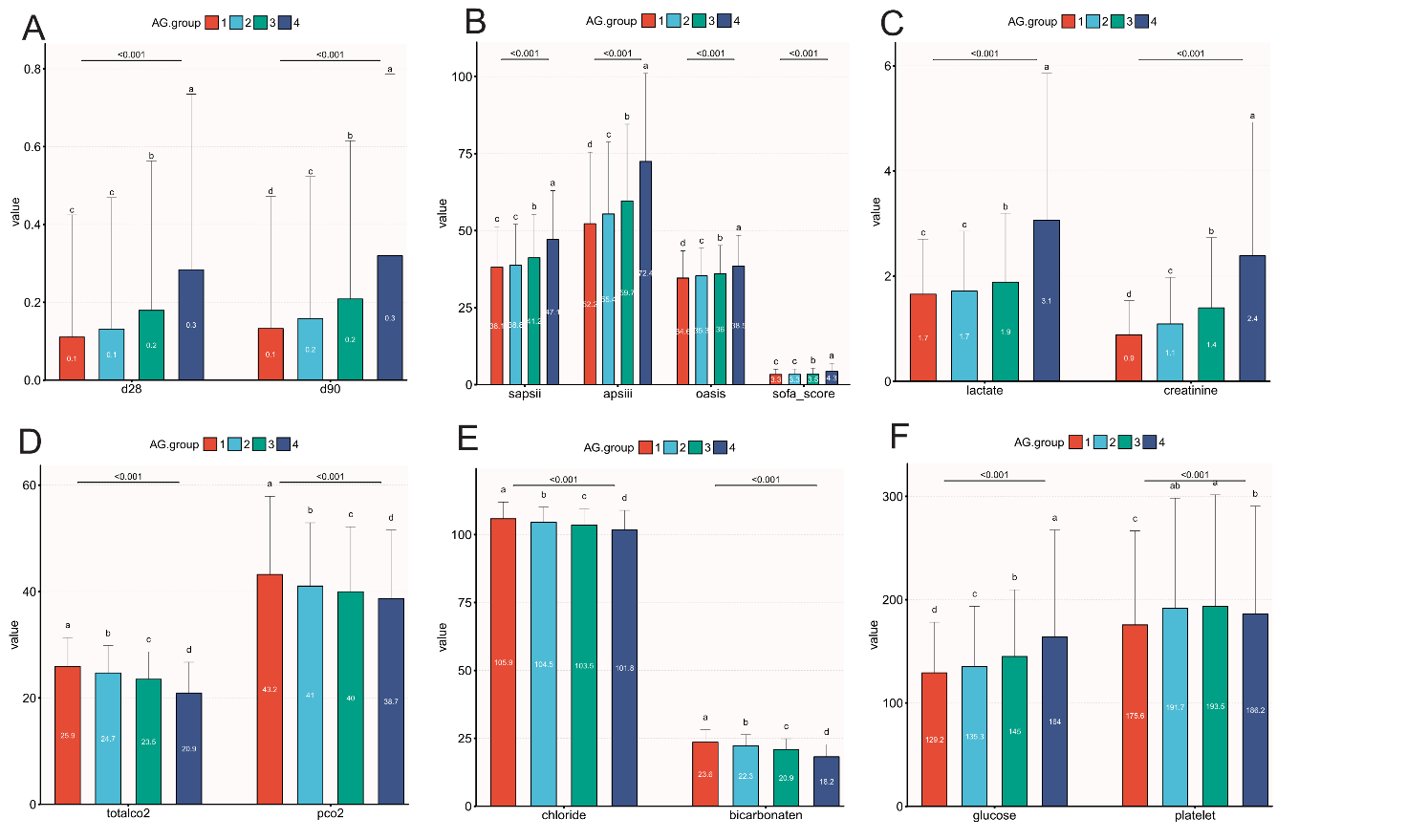


**Supplementary figure 2**. The positive association between the serum AG and lactate. A linear relationship was found between the AG and the serum lactate line curve fit (P for linearity<0.001).


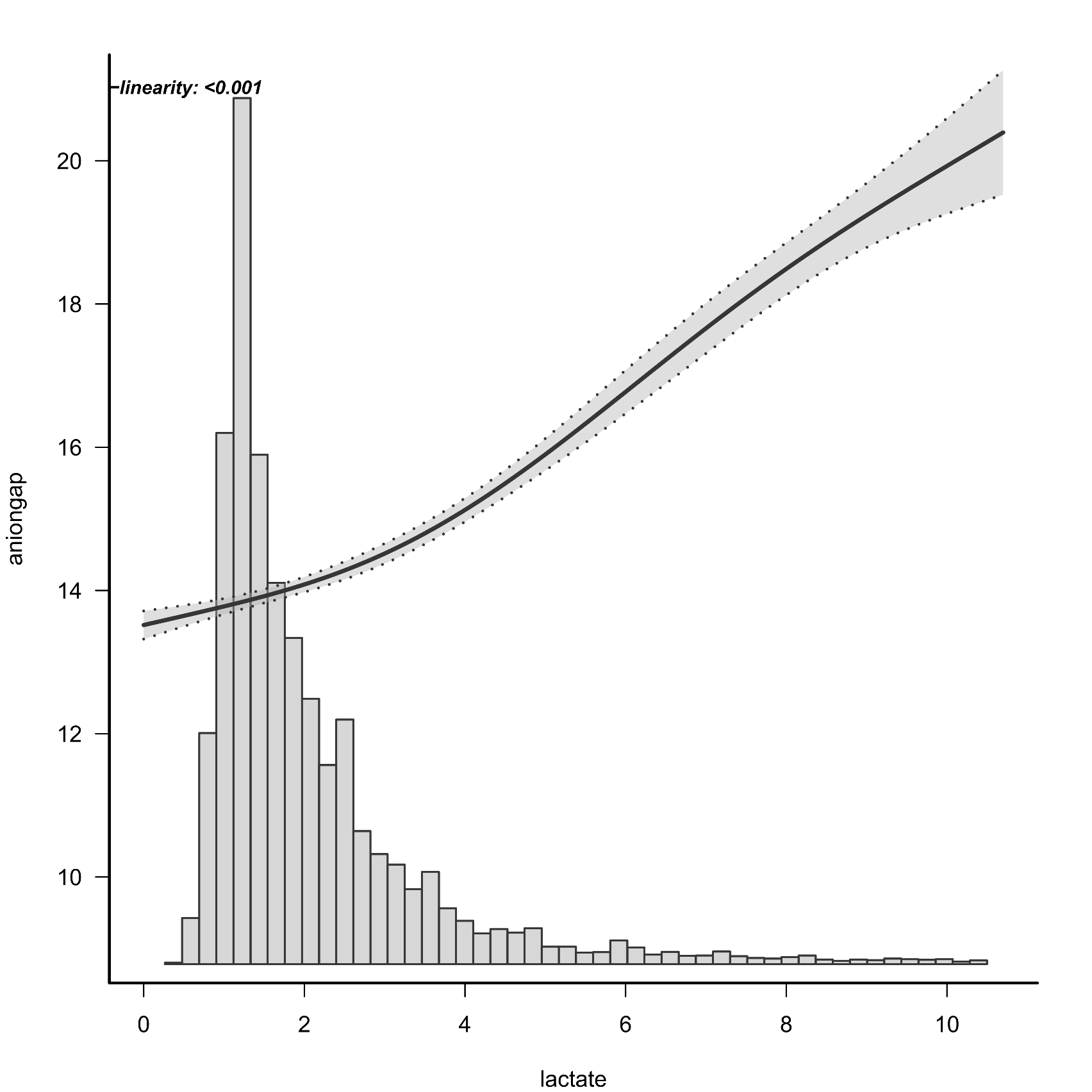


**Supplementary figure 3.** The analysis of E-value for unmeasured confounding.


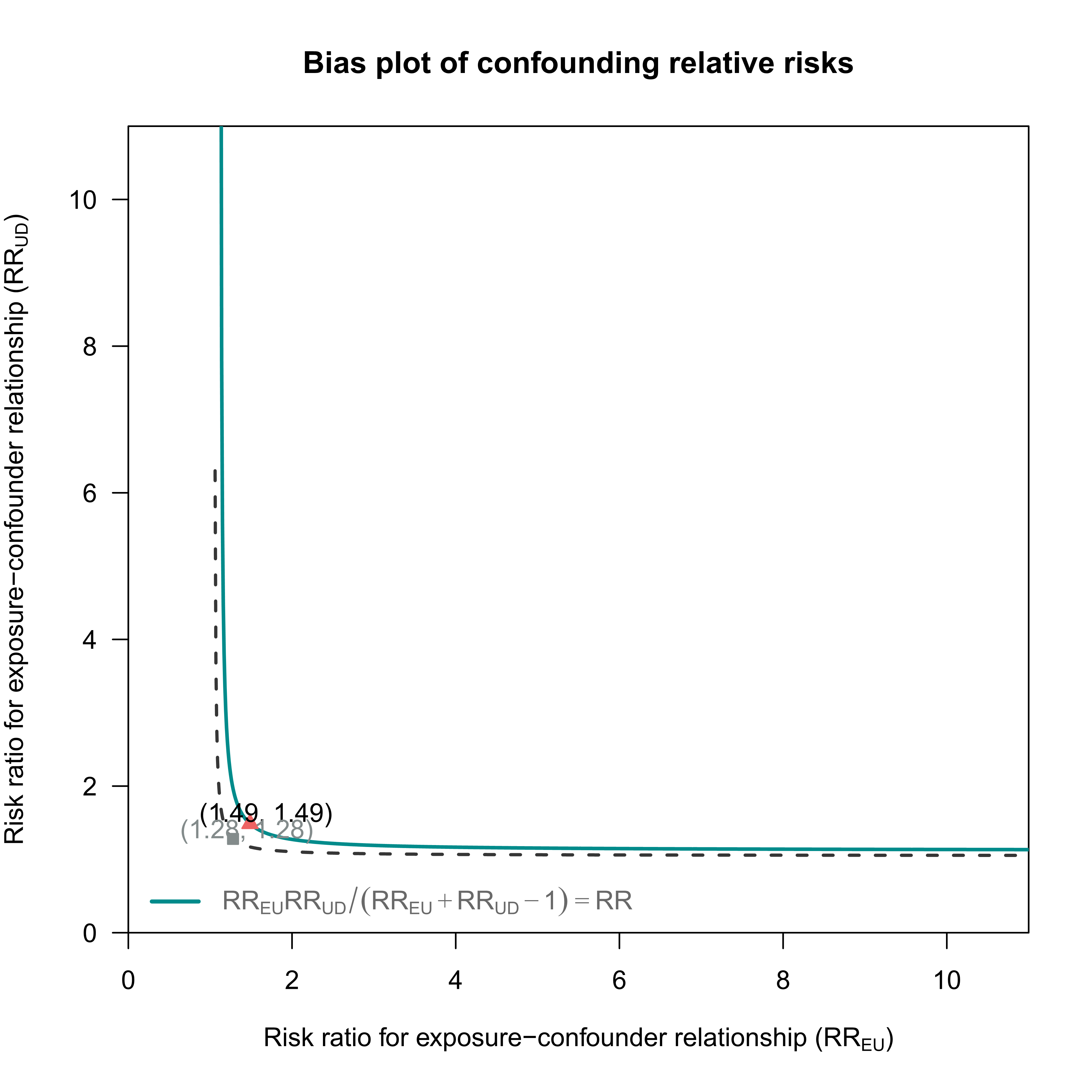

Supplement: Supplementary file 1 [file medi-103-e39029-s001.docx]
